# Idiosyncratic gesture use in a mother-infant dyad in chimpanzees (*Pan troglodytes schweinfurthii*) in the wild

**DOI:** 10.1007/s10071-024-01904-3

**Published:** 2024-10-03

**Authors:** Bas van Boekholt, Isabelle Clark, Nicole J. Lahiff, Kevin C. Lee, Katie E. Slocombe, Claudia Wilke, Simone Pika

**Affiliations:** 1 Comparative BioCognition, Institute of Cognitive Science, Osnabrück University, Osnabrück, Germany; 2 Department of Anthropology, University of Texas at Austin, Austin, TX United States of America; 3 Department of Psychology, University of York, York, UK; 4 Department of Evolutionary Anthropology, University of Zurich, Zurich, Switzerland; 5 Department of Comparative Linguistics, University of Zurich, Zurich, Switzerland; 6 School of Human Evolution and Social Change, Arizona State University, Phoenix, AZ USA; 7 Institute of Human Origins, Arizona State University, Phoenix, AZ USA

**Keywords:** Idiosyncratic gestures, Gesture acquisition, Chimpanzees, Gestures, Mother-infant interactions, Evolution of language

## Abstract

One promising method to tackle the question, “In which modality did language evolve?” is by studying the ontogenetic trajectory of signals in human’s closest living relatives, including chimpanzees (*Pan troglodytes)*. Concerning gestures, current debates centre on four different hypotheses: “*phylogenetic ritualization*”, “*social transmission through imitation*”, “*ontogenetic ritualization*”, and “*social negotiation*”. These differ in their predictions regarding idiosyncratic gestures, making such occurrences a crucial area of investigation. Here, we describe a novel and potential idiosyncratic behaviour — ‘hand-on-eye’ — which was initially observed in one mother-infant dyad in a community of chimpanzees living in the wild. We systematically investigated the form, sequential organisation, intentionality, usage, function, and distribution of the behaviour over a five-year period. The results showed that ‘hand-on-eye’ was nearly exclusively deployed in a single mother-infant dyad, was accompanied by hallmarks of intentionality, and served to initiate or resume joint dorsal travel. Although the behaviour was observed once in each of three other mother-infant dyads, these lacked the same frequency and hallmarks of intentionality. ‘Hand-on-eye’ thus qualifies as an idiosyncratic gesture. The proposed developmental pathway gives support to both the “*ontogenetic ritualization*” and “*social negotiation*” hypotheses. It also stresses the crucial need for longitudinal approaches to tackle developmental processes that are triggered by unique circumstances and unfold over relatively long time windows.

**Supplementary Information:**

The online version contains supplementary material available at 10.1007/s10071-024-01904-3.

## Introduction

Language has often been suggested as one of the defining characteristics separating humans from the rest of the animal kingdom (Christiansen & Kirby 2003; Hauser et al. 2014; Pinker 1994). One crucial method to unravel the origins of language is the comparative approach investigating the behaviour of living and often closely related species to draw inferences about evolutionary trajectories (Fitch 2005, 2017; Pika 2015; van Horik & Emery 2011). While early comparative investigations into language origins have predominantly focused on vocalizations (Marler 1976; Struhsaker 1967; Winter et al. 1973), language is an integrated system of speech and gesture (Kendon 2000; McNeill 1985), with gestures defined as movements and body postures that are mechanically ineffective, directed to a recipient, and potentially elicit a voluntary response (Aychet et al. 2021; Fröhlich & Hobaiter 2018; Pika 2008a). Research into gestural signalling of other animals, specifically great apes, has increased considerably during the last decades, showing key similarities with language (Hobaiter & Byrne 2011; Hobaiter et al. 2022; Pika 2008a; Pika et al. 2005; Plooij 1978; Sievers et al. 2017; Tomasello & Call 2018).

However, how gestures are acquired during ontogeny remains contentious and multiple hypotheses have been proposed (Byrne et al. 2017; Liebal et al. 2019; Pika & Fröhlich 2019). The *phylogenetic ritualization hypothesis* postulates that gestures are innate and evolved from action sequences that previously had no communicative function (Byrne et al. 2017; Darwin 1872). Through ritualization over evolutionary time, these action sequences were shortened into communicative gestures. Alternatively, the *social transmission through imitation hypothesis* argues that gestures are learned within the lifetimes of individuals (Liebal & Call 2012; Tomasello et al. 1994). Individuals recognize the communicative intention of a gesturing individual and subsequently engage in imitation when they have the same communicative intention. The *ontogenetic ritualization hypothesis* suggests that existing action sequences shorten into communicative gestures through repeated interactions between the same two individuals (Bates et al. 1979; Tomasello & Call 2018; Tomasello et al. 1997; Vygotsky 1978). One example of this ritualization starts with an infant climbing on the mothers back to be carried (Pika & Fröhlich 2019; Tomasello & Call 2018). Over repeated interactions the mother facilitates this carrying by lowering her back as soon as the infant starts climbing. Subsequently, the infant will anticipate the mother lowering her back and only produces the initial part of climbing, touching the mother’s back. Here the action sequence of an infant climbing on the mother’s back is shortened into a “touch back” gesture followed by the mother lowering her back. Another hypothesis for gesture acquisition recently revised by Pika and Fröhlich is the *social negotiation hypothesis* (Fröhlich et al. 2016; Pika & Fröhlich 2019; Plooij 1978, 1984; Wittgenstein 1953). Like the *ontogenetic ritualization hypothesis,* it proposes that gestures are acquired within an individual’s lifetime through a social learning process. However, rather than gestures always stemming from full action sequences that shorten over repeated exchanges, it posits that gestures emerge from an exchange of social behaviours between interactants, resulting in mutual understanding that specific behavioural patterns can be used as communicative signals. Going back to the previous example of the “touch back” gesture, the *social negotiation hypothesis* posits that this gesture could also have originated from the infant touching the mother without any communicative intent. Over repeated exchanges the mother and infant negotiate a mutual understanding about the communicative meaning of these touches to facilitate carrying. In contrast to ontogenetic ritualization, individuals learn and attribute communicative meanings to specific gestures and can directly use this knowledge in interactions with unfamiliar partners. For a more comprehensive discussion of the distinctions among these four hypotheses, see Liebal and colleagues (2019).

One way to disentangle these different hypotheses is to focus on idiosyncratic gestures, which are only produced by one individual or dyad, as the hypotheses make different predictions about the occurrence of such idiosyncratic gestures (Call & Tomasello 2007; Pika & Fröhlich 2019; Tomasello et al. 1994; see Table 1). The *phylogenetic ritualization hypothesis* predicts an absence of idiosyncratic gestures. While social experience can determine the production of a gesture from the innate repertoire (i.e. the right circumstances must exist for them to be produced) and an individual may refine their repertoire leading to moderate variability within and between communities, *phylogenetic ritualization* does not allow for the formation of unique gestures (Amici & Liebal 2023; Liebal & Call 2012). The *social transmission through imitation hypothesis* likewise does not allow for the prolonged presence of idiosyncratic gestures, as any initially idiosyncratic gesture would be expected to spread throughout the community (Hobaiter & Byrne 2010; Liebal & Call 2012; Pika 2008b; Tomasello 1999). Conversely, the *ontogenetic ritualization hypothesis* and *social negotiation hypothesis* both predict a high degree of variation in gesture repertoires and the occurrence of idiosyncratic gestures (Liebal & Call 2012; Pika & Fröhlich 2019; Tomasello & Call 2018). Furthermore, longitudinal investigations can elucidate the unique set of circumstances that lead to the formation of idiosyncratic gestures within individuals (Howard et al. 2012). For example, they can reveal whether an idiosyncratic gesture started as an action sequence – i.e., ontogenetic ritualization – or its gestural form but without communicative meaning – i.e., social negotiation. Whilst these four hypotheses may not be mutually exclusive as different mechanisms might be involved for different gesture types (Bard et al. 2014; Liebal et al. 2019; Prieur et al. 2020; Tomasello & Call 2018), examining potential cases of idiosyncratic gesturing can shed light upon which processes have contributed to the acquisition of these specific gestures.

**Table 1:** Predictions for the presence of idiosyncratic gestures and their origins for the four hypotheses on gesture acquisition

Hypothesis	Presence of idiosyncratic gestures	Idiosyncratic gestures emerge from…
Phylogenetic ritualization	Absent	-
Social transmission through imitation	Absent over prolonged time periods	-
Ontogenetic ritualization	Present	A shortening of action sequences
Social negotiation	Present	Action sequences or gestural forms without communicative meaning

Initial studies focusing on gestural use of great apes reported relatively high degrees of idiosyncrasy (Call & Tomasello 2007; Pika et al. 2005; Tomasello et al. 1994). However, some scholars suggested that differences in gestural repertoires of the studied species and groups were premature assumptions, which could be due to limited sampling effort or differences in housing and living conditions (Genty et al. 2009; Hobaiter & Byrne 2011; Liebal et al. 2019). Notably, observation time was a strong predictor for an individual’s repertoire size, suggesting that apparent idiosyncrasy can be caused by under-sampling (Byrne et al. 2017; Hobaiter & Byrne 2011). Longitudinal studies on gestural ontogeny in great apes that span multi-year time periods are rare (Bard 1992; Bard et al. 2014; Fröhlich & Pika 2019; Plooij 1984; Tanner et al. 2006; Tomasello et al. 1997; van de Rijt-Plooij & Plooij 1987).

In this paper, we describe a novel potential idiosyncratic gesture we labelled ‘hand-on-eye’, occurring in a large community of chimpanzees living in their natural environment. The term ‘hand-on-eye’ refers to an individual deliberately placing one hand in front of the eye of another individual, blocking at least part of their visual field. After observing this behaviour in an infant chimpanzee, we collected detailed data on this behaviour over a five-year period on multiple mother-infant dyads to investigate its production frequency, distribution, variability, function and manifest intentionality as well as its potential developmental pathway (Bard et al. 2019; Wilke et al. 2022). To examine whether the behaviour qualifies as a gesture and, more specifically, an idiosyncratic one, we investigated 1) the ‘hand-on-eye’ movement sequential organisation, its form, and whether its production met markers of intentionality by performing detailed analyses on video materials of its occurrence; 2) its emergence in the initial observed infant from a longitudinal dataset, alongside its prevalence in the study population among dyads similar in age and context; 3) its usage and function by collecting systematic focal data on how often and in which contexts the movement appears, and the outcome it elicited. If ‘hand-on-eye’ qualifies as an idiosyncratic gesture, we would expect to find intentional, goal-directed, exclusive usage in one individual or dyad. If ‘hand-on-eye’ were acquired through ontogenetic ritualization we would expect to find evidence for an initial action sequence from which ‘hand-on-eye’ became ritualized; for example, the infant grabbing the mother’s head to “steer” her towards a goal. If ‘hand-on-eye’ were acquired through social negotiation we would expect to find initial use of this gestural form without communicative meaning, which then could be transferred to other individuals.

## Methods

### Study site and subjects

**Fig. 1:**
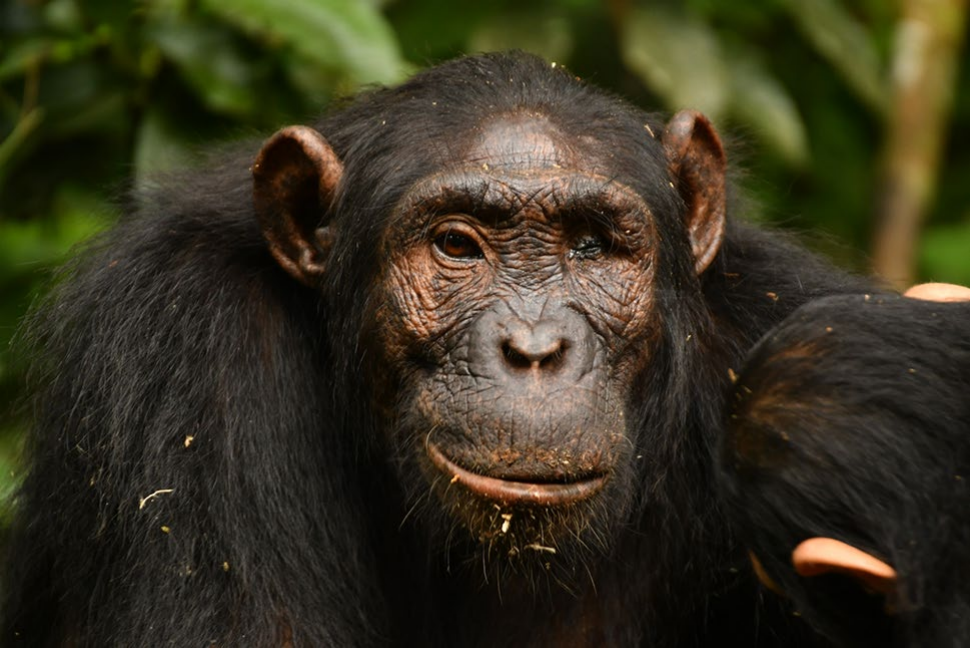
Beryl

Data were collected from the Ngogo chimpanzee community in Kibale National Park, Uganda between 2018 and 2023 via continuous focal-sampling (Altmann 1974) on a handheld device using HanDBase (v4.9.086, DDH software). Video data were collected with Panasonic HC-VX980 (2018–2020), Sony AX100E 4 K (2021–2023), and Panasonic HC-VX1 4 K cameras (2023). ‘Hand-on-eye’ was initially observed in the mother-infant dyad Beryl and Lindsay. Beryl immigrated into the study community in 2012, already missing her left eye (Fig. 1).

### Data analyses

- 1) Descriptive analysis of ‘hand-on-eye’
  To establish the sequential organisation, form, and intentionality involved in the production of ‘hand-on-eye’ within an interaction, we performed a descriptive analysis of all video-recorded instances (*n* = 21) of ‘hand-on-eye’ between Beryl and Lindsay. We showcase the sequential organisation of one representative episode, taking a conversation analytic approach (Fröhlich 2017; van Boekholt et al. 2024; Wilkinson et al. 2012). However, the specific forms of interactions involving ‘hand-on-eye’ varied on aspects such as starting arrangement, order of operations, hand used, eye covered, duration of the initial cover, response of mother, and behavioural outcome (see Table 2). Intentionality criteria included *persistence* and *elaboration*, defined as the production of the same gesture (persistence) or another signal (elaboration), including a change of eye covered, after *response waiting* (Graham et al. 2019; Rodrigues & Fröhlich 2021; see Table S1 for definitions). *Response waiting* on its own was not considered sufficient to establish intentionality as it is not possible to reliably distinguish between an individual simply abandoning the communicative attempt and “waiting” for a response (Ben Mocha & Burkart 2021; Townsend et al. 2017). Other established intentionality criteria such as *social use, attention-getting behaviours* and *sensitivity to recipient’s attentional state* were also not considered as they are less applicable due to the tactile nature of ‘hand-on-eye’ (Rodrigues & Fröhlich 2021). Both *persistence* and *elaboration* rely on the absence of an immediate satisfactory response of the recipient and, as such, could not be measured in all interactions (*n* = 8).
- 2) Prevalence of ‘hand-on-eye’ in the study population
  To explore the prevalence of ‘hand-on-eye’ throughout the population, we analysed video footage collected over a five-year period distributed over four field seasons (April 2018 – March 2020; December 2020 – September 2021; August 2022 – February 2023; March 2023 – September 2023) of a total of 12 mother-infant dyads with infants similar in age to Lindsay, including Beryl and Lindsay (Table S2). A total of 1203 mother-infant interactions, defined as any exchanges of signals and actions between the infant and its mother, were analysed for the occurrence of ‘hand-on-eye’ in nine different contexts (Table S3). To track the emergence of ‘hand-on-eye’, we considered interactions between Beryl and Lindsay in three quasi-continuous blocks separately (age Lindsay first block 3–25 months; second block 37–42 months; third block 56–68 months).
- 3) Systematic focal follows of Beryl and Lindsay
  To determine the usage and function of ‘hand-on-eye’ between Beryl and Lindsay, we systematically collected focal data on the behaviour of Lindsay in addition to the before-mentioned video footage. Focal data includes a total of 12.8 h collected on seven days from March to September 2023 during focal follows ranging in duration from 0.5 to 4.5 h. During focal follows, we recorded all occurrences of ‘hand-on-eye’ as well as additional data about the behavioural context in which ‘hand-on-eye’ occurred and the behavioural change of the recipient.

**Table 2:** Overview of variability in ‘hand-on-eye’ interactions. The first 21 interactions are between Beryl and Lindsay. The last row summarizes each column where the variations involving individuals other than Beryl and Lindsay are mentioned after the colon

Interaction number	Starting arrangement	Order of move dorsal (MD) vs. hand-on-eye movement (HOE)	Hand used with initial hand-on-eye movement	Eye covered with initial hand-on-eye movement	Duration initial hand-on-eye movement in seconds	Response mother to initial hand-on-eye movement	Behavioural outcome	Infant age in months
1 (Beryl – Lindsay)	Ventral—Ventral	MD → HOE	Right	Right	< 0.5	No response	Nothing	42
2	Distal (1.5 m)	MD → HOE	Right	Right	< 0.5	No response	Nothing	42
3	Distal (2 m)	MD → HOE	Left	Left	1	Grabbing hand	Initiate playing	42
4	Ventral—Ventral	MD → HOE	Right	Right	Not visible	Getting up to travel	Initiate joint travel	56
5	Ventral (Infant) – Dorsal (Mother)	MD → HOE	Right	Right	< 0.5	Turn head away	Initiate joint travel	56
6	Ventral (Infant) – Dorsal (Mother)	HOE → MD	Right	Right	1.5	Turn head away	Initiate joint travel	56
7	Side-by-side	HOE → MD	Left (right not in reach)	Left	< 0.5	Getting up to travel	Initiate joint travel	59
8	Ventral—Ventral	HOE → Move ventral	Left	Right	1	Getting up to travel	Initiate joint travel	59
9	Side-by-side	NA (no move dorsal)	Right	Right	1	No response	Food sharing	59
10	Ventral (Infant) – Dorsal (Mother)	MD → HOE	Left (might not have been first HOE)	Left (might have been preceded by right eye but not visible)	1.5	Getting up to travel	Initiate joint travel	59
11	Distal (1 m)	HOE → MD	Left	Right	0.5	Getting up to travel	Initiate joint travel	60
12	Ventral—Ventral	HOE → MD	Left	Left	< 0.5	Reposition body	Initiate joint travel	60
13	Ventral (Infant) – Dorsal (Mother)	MD → HOE	Right	Left	< 0.5	No response	Initiate joint travel	60
14	Ventral (Infant) – Dorsal (Mother)	HOE → MD	Right	Right	< 0.5	No response	Initiate joint travel	63
15	Ventral (Infant) – Dorsal (Mother)	NA (Dorsal from start clip)	Right	Right	< 0.5	No response	Initiate nursing	63
16	Dorsal riding	NA (Dorsal from start clip)	Left	Right	< 0.5	Turn head away	Restart joint travel	68
17	Dorsal riding	NA (Dorsal from start clip)	Left	Right	< 0.5	Turn head away	Restart joint travel	68
18	Side-by-side	MD → HOE	Right	Right	< 0.5	Getting up to travel	Initiate joint travel	68
19	Side-by-side	NA (no move dorsal)	Right	Right	1	No response	Initiate nursing	68
20	Dorsal riding	NA (Dorsal from start clip)	Right	Right	< 0.5	Restart joint travel	Restart joint travel	68
21	Dorsal riding	NA (Dorsal from start clip)	Left (might have not been first HOE)	Left (might have not been first HOE)	2	No response	Restart joint travel	68
22 (Miliah – Malaika)	Side-by-side	NA (no move dorsal)	Left	Right	< 0.5	Turn head towards infant	Food sharing	31
23 (Sabin – Louis)	Side-by-side	NA (no move dorsal)	Right	Right	2	Turn head away	Initiate nursing	43
24 (Violetta – Hubble)	Ventral (Infant) – Dorsal (Mother)	MD → HOE	Right	Right	1	Turn head away	Initiate joint travel	42
Total	Ventral – Ventral (4); Distal (3); Ventral – Dorsal (6:1); Side-by-side (4:2); Dorsal riding (4)	MD → HOE (8:1); HOE—> MD (5); HOE—> MV (1); NA (7:2)	Left (6:1); Left* (3); Right (12:2)	Left (4); Left* (2); Right (15:3)	< 0.5 s (12:1); > 0.5 s (8:2); NA (1)	No response (8); Getting up to travel (6); Turn head away (4:2); Other response (3:1)	Initiate joint travel (11:1); Restart joint travel (4); nothing (2); Initiate nursing (2:1); Food sharing (1:1); Initiate playing (1)	

## Results

**Fig. 2:**
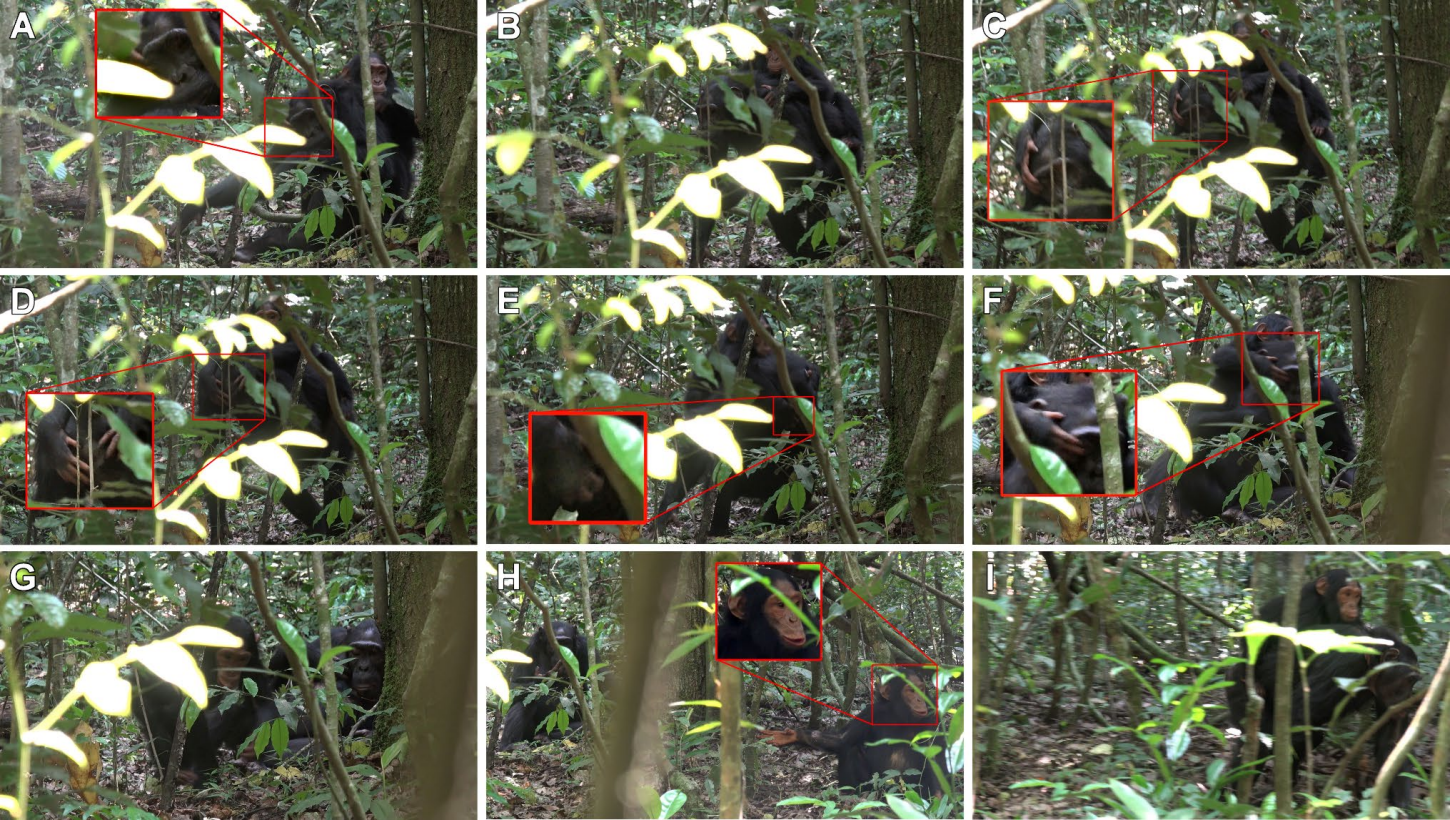
Screenshots of key moments from exemplar sequence of use of ‘hand-on-eye’ during a joint-travel initiation between Lindsay and Beryl. Complete description found in Results, and the full video clip is included in the Supplemental Materials. **A** – **C**: Two instances of ‘hand-on-eye’ in rapid succession from Lindsay as Beryl gets up after resting. **D**: Beryl turns head away from Lindsay’s right hand. Lindsay persists with minor elaboration by reaching both hands to cover both of Beryl’s eyes. **E**: Beryl turns further away from Lindsay’s hands. Lindsay persists yet again with a very extended reach to cover Beryl’s right eye. **F**. Lindsay reaching to cover Beryl’s right eye upon re-approaching after walking away briefly from Beryl. **G**: Lindsay walks away from Beryl again, with a POUT FACE, while emitting quiet WHIMPER vocalizations. **H**: Lindsay EXTENDS HAND towards Beryl, while WHIMPERING with a POUT FACE from ~ 5 m away. **I**: Lindsay mounted dorsally on Beryl after joint-travel begins

- 1) Descriptive analysis of ‘hand-on-eye’
  Here, we describe the sequential organisation of an episode between Beryl and Lindsay as an archetypic example of when and how Lindsay displays ‘hand-on-eye’, including both *persistence* and *elaboration* (full video clip in the Supplemental Materials, interaction number 5 in Tables 2 and 3). Gestures previously described in the existing literature on chimpanzee communication (Fernandez-Carriba et al. 2002; Goodall 1986; Nishida et al. 1999; see Table S4) are denoted in capitals.
  At the start, Beryl is lying down while Lindsay sits behind, grooming Beryl. After ~ 5 s, Lindsay stops grooming and Beryl rises — first to a sitting then into a quadrupedal standing position. As Beryl rises, Lindsay climbs onto Beryl’s back while performing **hand-on-eye** using her right hand to cover Beryl’s right eye twice within a second (Fig. 2, A-C). Beryl responds by turning her head to the left, moving the right side of her face out of Lindsay’s current reach, thereby ending Lindsay’s eye cover. Directly after this head movement, Lindsay then shows *elaboration* by extending both of her hands to **cover both of Beryl’s eyes** (Fig. 2, D). Beryl then turns her whole body to the left after which Lindsay shows *persistence* by performing another **hand-on-eye**, greatly extending her right hand to reach around Beryl’s bowed head to cover Beryl’s right eye (Fig. 2, E). This eye cover lasts for ~ 2 s as Beryl moves back to a lying position. Lindsay dismounts and walks ~ 1 m away. Lindsay then pauses her movement for ~ 3 s, during which Beryl rises into a sitting position. Sensing no further movement from Beryl, Lindsay re-approaches. While moving around to mount dorsally on Beryl, Lindsay *persists* again by performing another **hand-on-eye** using her right hand to cover Beryl's right eye (Fig. 2, F). Beryl responds by going back into a lying position while self-scratching and self-grooming. Lindsay walks away from Beryl again, *elaborating* on her earlier attempts by performing WHIMPER vocalizations and displaying a POUT FACE (Fig. 2, G). From ~ 5 m away, Lindsay stops moving and looks back at Beryl. Lindsay then turns around, sits down, and displays an EXTEND HAND gesture, all the while continuing her WHIMPER vocalizations and POUT FACE (Fig. 2, H). This goes on for ~ 13 s with Lindsay directing her gaze to either Beryl or the observer. Finally, Lindsay rises and walks back towards Beryl. Beryl also rises and moves towards Lindsay, who pauses halfway and waits. As Beryl passes Lindsay, Lindsay climbs into a dorsal mount position, keeping her hands in a neutral position away from Beryl’s eyes and head while Beryl continues walking (Fig. 2, I).
  Across all video-recorded episodes exhibiting ‘hand-on-eye’ between Beryl and Lindsay, 15 of 21 (71.43%) instances occurred during some form of joint-travel, which can be further subdivided into initiating joint-travel (*n* = 11) and resuming joint-travel (*n* = 4) (Table 2). The remaining instances occurred during affiliation (*n* = 2), nursing (*n* = 2), feeding (*n* = 1), and playing (*n* = 1) contexts. Intentionality criteria were detected in 7 of the 13 (53.85%) instances when no immediate satisfactory response was given, with both *persistence* and *elaboration* often appearing in conjunction (*n* = 6; see Table 3).
- 2) Prevalence of ‘hand-on-eye’ in the study population
  There were 46 instances of ‘hand-on-eye’ distributed over 24 interactions recorded in 1203 interactions (2.00%) across 12 age-matched mother-infant dyads. These instances were distributed over four different dyads with ‘hand-on-eye’ occurring once each in three dyads and the rest occurring between Beryl and Lindsay (21/24 = 87.50%; see Table 4). ‘Hand-on-eye’ was produced in five different contexts with the highest frequency appearing in the joint-travel context (16/24 = 66.67%; see Table 4). ‘Hand-on-eye’ was only produced by Lindsay after she had reached three-and-a-half years of age and only produced in the joint-travel context after she reached four-and-a-half years of age. ‘Hand-on-eye’ was seen in multiple dyads, including in the two dyads with the highest sampling effort (Table 4), indicating that it is performed by others and its detection may be related to sampling effort. However, its use between Beryl and Lindsay has certain defining features not seen in other dyads. Its gestalt, with the infant covering the eye from a dorsal position over the head of the mother, only appeared in a single instance in one other dyad, where three-year-old Hubble used it on his mother, Violetta, and they subsequently started joint-travel (Table 2). Whilst Lindsay showed intentional production in the form of *persistence* and/or *elaboration* of the ‘hand-on-eye’ in 54% of instances when an immediate response was not obtained, this was not observed in Hubble’s case (the mothers responded immediately in Malaika's and Louis’s cases; Table 3).
- 3) Systematic focal follows of Beryl and Lindsay
  During focal following from March – September 2023, Lindsay was observed to produce ‘hand-on-eye’ 29 times. These instances occurred exclusively in joint-travel interactions and were distributed over 15 joint travel bouts (1.9 ± 1 instances per bout) representing roughly a quarter of all observed joint-travel interactions (15/58 = 25.9%). In these interactions, ‘hand-on-eye’ either led to the initiation of a joint-travel (*n* = 6) or were produced after Beryl stopped moving (*n* = 9), sometimes leading to resumption of travel (*n* = 5).

**Table 3:** Overview of the occurrence of different intentionality criteria in ‘hand-on-eye’ interactions. The first 21 interactions are between Beryl and Lindsay. Persistence or elaboration where only possible if the recipient did not show any response within two seconds and after response waiting, denoted by NA if this was not the case. The last row summarizes each column where the cases involving individuals other than Beryl and Lindsay are mentioned after the colon

Interaction number	Leads to joint travel?	Persistence	Elaboration
1 (Beryl – Lindsay)	No (no apparent outcome)	NA	NA
2	No (no apparent outcome)	Yes	Yes (Change of eye; Both eyes)
3	No (playing)	NA	NA
4	Yes (< 2 s)	NA	NA
5	Yes	Yes (2)	Yes (Change of eye; Present; Extend hand; Whimper; Pout face)
6	Yes	NA	NA
7	Yes (< 2 s)	NA	NA
8	Yes (< 2 s)	NA	NA
9	No (food sharing)	NA	NA
10	Yes	NA	NA
11	Yes (< 2 s)	NA	NA
12	Yes	Yes (9)	Yes (Change of eye)
13	Yes (< 2 s)	NA	NA
14	Yes	Yes (2)	Yes (Change of eye)
15	No (nursing)	NA	NA
16	Yes (< 2 s)	NA	NA
17	Yes (< 2 s)	NA	NA
18	Yes	Yes	Yes (Exaggerated loud scratch; push; possible change of eye)
19	No (nursing)	Yes (1)	Yes (Whimper; Extend hand)
20	Yes (< 2 s)	NA	NA
21	Yes	No	Yes (Change of eye)
22 (Miliah – Malaika)	No (food sharing)	NA	NA
23 (Sabin – Louis)	No (nursing)	NA	NA
24 (Violetta – Hubble)	Yes	No	No
Total	Yes (< 2 s) (8); Yes (7:1); No (6:2)	Yes (6); No (1:1); NA (14:2)	Yes (7) – Change of eye (5); Both eyes (1); Extend hand (2); Exaggerated loud scratch (1); Whimper (2); Both eyes (1); Present (1); Pout face (1) – No (0:1); NA (14:2)

**Table 4:** Overview of all occurrences of ‘hand-on-eye’ sorted by context and dyad. Number in parentheses is the total number of interactions analysed for that combination of context and dyad. To investigate the emergence of ‘hand-on-eye for Beryl and Lindsay data for this dyad were split across the three quasi-continuous study periods. Occurrences of 'hand-on-eye' are displayed in bold

Dyads (infant age range in months)	Contexts									
	Affiliation	Feeding	Grooming	Nursing	Other	Playing	Resting	Travelling	Weaning	Total
Beryl – Lindsay (3 – 22)	0 (3)	0 (7)	0 (7)	0 (3)	0 (6)	0 (7)	0 (3)	0 (16)	–	0 (52)
Beryl – Lindsay (37 – 42)	**2 (2)**	0 (1)	0 (4)	0 (5)	–	**1 (4)**	0 (1)	0 (4)	–	**3 (21)**
Beryl – Lindsay (56 – 68)	0 (0)	**1 (2)**	0 (28)	**2 (25)**	–	0 (3)	–	**15 (48)**	–	**18 (106)**
Miliah – Malaika (25 – 49)	0 (8)	**1 (15)**	0 (43)	0 (29)	0 (3)	0 (13)	0 (3)	0 (123)	–	**1 (237)**
Baez – Camilla (8 – 28)	–	0 (2)	0 (2)	0 (2)	–	0 (1)	0 (2)	0 (12)	–	0 (21)
Fitzgerald – Gatsby (6 – 51)	–	0 (4)	0 (40)	0 (18)	0 (1)	0 (24)	–	0 (81)	–	0 (168)
Renata – Malala (6 – 58)	–	–	0 (12)	0 (2)	-	0 (1)	–	0 (13)	–	0 (28)
Violetta – Hubble (10 – 59)	–	0 (1)	0 (13)	0 (13)	0 (1)	0 (1)	–	**1 (26)**	–	**1 (55)**
Callas – Kano (12 – 56)	0 (1)	0 (10)	0 (19)	0 (9)	–	0 (8)	0 (8)	0 (33)	–	0 (88)
Fiona – Kofi (14 – 54)	–	0 (1)	0 (7)	0 (1)	–	0 (1)	–	0 (3)	–	0 (13)
Shire – Tolkien (6 – 38)	–	–	–	–	–	0 (3)	–	0 (9)	–	0 (12)
Sabin – Louis (6 – 60)	0 (5)	-	0 (100)	**1 (23)**	0 (2)	0 (3)	0 (2)	0 (46)	-	**1 (181)**
Rusalka – Dorothy (13 – 61)	-	-	0 (31)	0 (5)	-	0 (6)	-	0 (17)	-	0 (59)
Atwood – Gunnel (12 – 44)	-	-	0 (1)	0 (2)	-	0 (4)	0 (1)	0 (11)	-	0 (19)
Carson—E.O. (8 – 64)	0 (1)	0 (5)	0 (54)	0 (25)	0 (3)	0 (5)	0 (3)	0 (42)	0 (5)	0 (143)
Total	**2 (20)**	**2 (48)**	0 (361)	**3 (162)**	0 (16)	**1 (84)**	0 (23)	**16 (484)**	0 (5)	**24 (1203)**

## Discussion

In this study, we investigated a novel and potentially idiosyncratic gesture used in a wild community of chimpanzees. Both the results from the video recordings as well as the systematic focal follows showed a consistent production of ‘hand-on-eye’ (65 instances divided over 33 interactions) from Lindsay towards her mother Beryl spanning multiple years. Its use was accompanied by several markers of intentional production over multiple instances, as well as a specific sequential organisation. Concerning usage and function, the behaviour was predominantly used to initiate joint dorsal travel, or, when already dorsal travelling, to resume travel. ‘Hand-on-eye’ was not exclusive to Beryl and Lindsay and was performed on singular occasions by three other infants, for two of whom more video footage were collected compared to Lindsay and Beryl (Table 3). Lindsay’s production appears unique in its repeated and intentional usage. More instances of ‘hand-on-eye’ might have been identified had we had greater sampling effort with other dyads. However, upon reviewing a substantial body of interactions from 11 other infants interacting with their mothers at similar ages to Lindsay we found minimal evidence for similar usage, suggesting ‘hand-on-eye’ is likely an idiosyncratic gesture in this population.

The hand-on-eye gesture has not been formerly documented in the gestural repertoire of chimpanzees (Call & Tomasello, 2007; Hobaiter & Byrne 2011; Nishida et al. 1999; Roberts et al. 2012) or other great apes (Fröhlich et al. 2021; Genty et al. 2009; Graham et al. 2017), and was mainly produced within one dyad. As such, the *phylogenetic ritualization hypothesis* does not explain the acquisition of ‘hand-on-eye’. ‘Hand-on-eye’ occurred primarily during a frequent social behaviour – joint-travel in a mother-infant dyad. Therefore, it seems unlikely that the signal has not previously been selected by other individuals from a larger innate repertoire, both within this study sample and other formerly studied groups, given its apparent effectiveness as exhibited by Lindsay and Beryl.

Our longitudinal dataset on Lindsay provides indications of the time period over which the hand-on-eye gesture and its intentional use developed. Whilst no recorded cases of ‘hand-on-eye’ were identified when Lindsay was aged zero to two years, by the age of three-and-a-half, Lindsay was first observed to use ‘hand-on-eye’. These initial uses did not lead to joint dorsal travel, but, at the age of four-and-half years Lindsay uses ‘hand-on-eye’ regularly and successfully to initiate joint dorsal travel. How the gesture emerged exactly during this period is unknown. However, here we are suggesting one possible developmental pathway for ‘hand-on-eye’. During early dorsal travel episodes, Lindsay could have sometimes “accidentally” blocked Beryl’s eyesight. This could have been driven by the infant’s intention to change the mother’s behaviour, with the only available surface to act on being the mother’s shoulders or head region. While this initial eye covering can happen in all mother-infant dyads, as suggested by single occurrences we observed in other dyads, it may have elicited a stronger response from Beryl because of her missing eye. This may have encouraged Lindsay to produce it more often, leading to repeated exchanges and a mutual understanding of the gesture being related to travel. Later, Lindsay could then flexibly use this ‘hand-on-eye’ gesture to initiate joint-travel. This initial eye cover could also be considered an action sequence similar to how touching the back is proposed to be ritualized from an infant climbing on its mother’s back (Tomasello & Call 2018). This proposed pathway therefore provides support for both the *ontogenetic ritualization hypothesis* and the *social negotiation hypothesis.* The *social negotiation hypothesis* states that individuals can transfer their knowledge and gestural usage to dyadic interactions with other individuals in their groups (Pika & Fröhlich 2019). However, with joint dorsal travel being almost exclusive to mother-infant dyads, the potential for transfer of the ‘hand-on-eye’ gesture might be limited making it difficult to distinguish between the two hypotheses. Additionally, the proposed developmental pathway does not explain how we have a similar instance of ‘hand-on-eye’ in the dyad of Violetta and Hubble, even though Violetta has both eyes. Due to both dyads being in the same community, Hubble might have socially learned the gesture from observations of Lindsay, which would support the *social transmission through imitation hypothesis* (Liebal & Call 2012; Tomasello 1999). Similar observations were made in another chimpanzee community, where able-bodied individuals adopted a liana-scratch technique that originated in one handicapped individual (Hobaiter & Byrne 2010). Ultimately, the current data shows that the different hypotheses do not have to be mutually exclusive (Bard et al. 2014; Tomasello & Call 2018). ‘Hand-on-eye’ might have emerged between Beryl and Lindsay through *ontogenetic ritualization* or *social negotiation* and then spread to other dyads through *imitation*. Continued observations of this community would further our understanding here on two fronts. First, if Lindsay would use ‘hand-on-eye’ to initiate joint-travel with other individuals, it would provide support for the *social negotiation hypothesis*. Second, if the usage of the gesture spreads further within the group, it would provide additional support for the *social transmission through imitation hypothesis*.

The ‘hand-on-eye’ gesture completely blocks Beryl’s visual field when performed to her only remaining eye. The increased effectiveness of eye covering in this dyad might have played a role in the formation of this gesture. However, we cannot determine whether the gesture took hold simply because Beryl reacted more strongly than she would with two eyes – i.e. operant conditioning – or whether Lindsay was able to take the perspective of Beryl and understand that, by covering her one eye, she effectively blocked Beryl’s visual field – i.e., theory-of-mind (Bräuer et al. 2007, 2020; Hare et al. 2000). The data point to the former, although Lindsay displayed a preference for covering Beryl’s right eye (15/18, Table 2), she also sometimes covered Beryl’s left eye socket, both as *elaboration* and *persistence*, and before covering the right eye, even in instances where both eyes were within arms’ reach.

Idiosyncratic gestures reveal the unique set of circumstances under which new gestures can emerge, deepening our understanding of language evolution (Botha 2007; Morford 1996). We initially observed hand-on-eye in a single mother-infant dyad and examined it as a potential case of an idiosyncratic gesture. Detailed analysis revealed that while the gesture occurred mostly between Lindsay and Beryl to initiate joint travel, similar forms of the gesture were observed on singular occasions in three other dyads. We argue that Beryl’s missing eye might have fostered the emergence of this gesture by bolstering its effectiveness to change the mother’s behaviour. Our longitudinal investigation into the emergence of the ‘hand-on-eye’ gesture in Lindsay indicates that frequent, goal-directed use of the gesture developed over a two-year period and that the gesture was most likely acquired through processes predicted by the *ontogenetic ritualization* or the *social negotiation hypothesis*.

## Supplementary Information

Below is the link to the electronic supplementary material.

Supplementary file1 (DOCX 39 KB)

Supplementary file2 (MP4 543080 KB)

## Data Availability

The data associated with this research are available at: https://osf.io/zem28/?view_only=0f905fc10ac54046b5d8d335f71f3d79.
