# Supplementary material for: Idiosyncratic gesture use in a mother-infant dyad in chimpanzees (Pan troglodytes schweinfurthii) in the wild
Source: Anim Cogn. 2024 Oct 3;27(1):64. doi: 10.1007/s10071-024-01904-3 (PMC11450076; doi:10.1007/s10071-024-01904-3)
Supplement: Supplementary file 1 — Supplementary file1 (DOCX 39 KB) [file 10071_2024_1904_MOESM1_ESM.docx]

**Idiosyncratic gesture use in a mother-infant dyad in chimpanzees (*Pan troglodytes schweinfurthii*) in the wild**

**Bas van Boekholt (0000-0002-3399-6938)^1^, Isabelle Clark (0000-0001-9534-7944)^2^, Nicole Lahiff (0000-0002-7150-7226)^3,4,5^, Kevin C. Lee (0000-0002-5606-8683)^6,7^, Katie E. Slocombe (0000-0002-7310-1887)^3^, Claudia Wilke (0000-0001-6351-0524)^3^, and Simone Pika (0000-0002-4398-2337)^1^**

^1^ Comparative BioCognition, Institute of Cognitive Science, Osnabrück University, Osnabrück, Germany

^2^ Department of Anthropology, University of Texas at Austin, Austin, Texas, United states of America

^3^ Department of Psychology, University of York, York, United Kingdom

^4^ Department of Evolutionary Anthropology, University of Zurich, Switzerland

^5^ Department of Comparative Linguistics, University of Zurich, Switzerland

^6^ School of Human Evolution and Social Change, Arizona State University, Phoenix, Arizona, United States of America

^7^ Institute of Human Origins, Arizona State University, Phoenix, Arizona, United States of America

*** Correspondence:**Corresponding Author
[basvanboekholt@hotmail.com](mailto:basvanboekholt@hotmail.com)

**Supplementary material**

*Table S1: Overview of analysed intentionality criteria, definitions and occurrences*

| **Intentionality criteria** | **Definition** | **Occurrence** |
| --- | --- | --- |
| Response waiting | Signaller pauses for at least two seconds after the production of the signal if no apparent satisfactory response is achieved. This was not considered an intentionality criterion on its own. | NA |
| Persistence | The production of the gesture is followed by more productions of the same gesture after response waiting | 6 |
| Elaboration | The production of the gesture is followed by more productions of other signals after response waiting, including the covering of the other eye | 7 |

Table S2: Overview of mother-infant dyads observed for the occurrence of hand-on-eye

| **Mother (estimated date of birth)** | **Infant (estimated date of birth)** | **Group** | **Number of ‘hand-on-eye’ seen (interactions analysed)** |
| --- | --- | --- | --- |
| Beryl (01-01-1999) | Lindsay (17-01-2018) | Central-East | 21 (179) |
| Atwood (02-07-1996) | Gunnell (31-12-2017) | Central-East | 0 (19) |
| Baez (01-11-2001) | Camilla (05-12-2018) | Central-East | 0 (21) |
| Callas (01-12-1985) | Kano (08-03-2018) | Central-East | 0 (88) |
| Carson (05-04-1994) | E.O. (05-12-2017) | West | 0 (143) |
| Fiona (01-01-1997) | Kofi (23-02-2018) | Central-East | 0 (13) |
| Fitzgerald (01-01-1996) | Gatsby (04-10-2018) | Central-East | 0 (168) |
| Miliah (01-09-2004) | Malaika (28-12-2018) | West | 1 (237) |
| Renata (01-03-2004) | Malala (23-03-2018) | West | 0 (28) |
| Rusalka (01-01-1990) | Dorothy (15-01-2018) | West | 0 (59) |
| Sabin (01-07-1997) | Louis (25-01-2018) | West | 1 (181) |
| Shire (01-01-1996) | Tolkien (02-02-2018) | Central-East | 0 (12) |
| Violetta (01-01-1981) | Hubble (18-03-2018) | Central-East | 1 (55) |

*Table S3: Overview of contexts analysed for the occurrence of the hand-on-eye gesture. Definitions are from established literature (Fraser et al., 2008; Goodall, 1986; Nishida et al., 1999; Pollick & de Waal, 2007)*

| **Context** | **Definition** | **Number of ‘hand-on-eye’ seen (interactions analysed)** |
| --- | --- | --- |
| Affiliation | Situations in which individuals are in non-agonistic contact such as embrace or gentle touch, with the exception of playing and grooming. | 2 (20) |
| Feeding | Situations in which individuals gather, process, or eat food; Interactions in which food is involved, such as begging or stealing, are included in this context. | 2 (48) |
| Grooming | Situations in which individuals use one or both hands to push aside hair of another individual | 0 (361) |
| Nursing | Situations in which the infant is attempting or actually suckling from the mothers’ nipple | 3 (162) |
| Other | Situations in which none of the other contexts accurately describe the behaviour. | 0 (16) |
| Playing | Situations in which individuals show non-adaptive, voluntary, “non-serious” behaviour such as, wrestle, chase, push and/or tickle a relaxed non-agonistic manner often accompanied with a ‘play face’ | 1 (84) |
| Resting | Situations in which individuals are sitting or lying down and for the most time not feeding, grooming or playing. Also includes self-grooming. | 0 (23) |
| Travelling | Situations in which individuals leave a location to locomote for prolonged bouts, either alone or following or leading other individuals. | 16 (484) |
| Weaning | Situations in which there is a nursing conflict between mother and infant. | 0 (5) |

*Table S4: Signals and definitions occurring in the described episode of ‘hand-on-eye’ between Beryl and Lindsay*

| **Signal** | **Definition** | **Reference** |
| --- | --- | --- |
| EXTEND HAND | The arm is reached out to the recipient with the palm held vertically or upwards and the fingers in an open position. | Nishida et al., 1999 |
| POUT FACE | Slightly open mouth in which the lips are pushed forward and rounded creating a small aperture. | Fernandez-Carriba et al., 2002 |
| WHIMPER | Highly tonal series of “Hoo-like” calls. | Goodall, 1986 |

**References**

Fernandez-Carriba, S., Loeches, A., Morcillo, A., & Hopkins, W. D. (2002). Asymmetry in facial expression of emotions by chimpanzees. *Neuropsychologia*, *40*(9), 1523-1533. <https://doi.org/10.1016/s0028-3932(02)00028-3>

Fraser, O. N., Schino, G., & Aureli, F. (2008). Components of relationship quality in chimpanzees. *Ethology*, *114*(9), 834-843. <https://doi.org/10.1111/j.1439-0310.2008.01527.x>

Goodall, J. (1986). *The chimpanzees of Gombe: Patterns of Behaviour*. Belknap Press of Harvard University Press.

Nishida, T., Kano, T., Goodall, J., McGrew, W. C., & Nakamura, M. (1999). Ethogram and ethnography of Mahale chimpanzees. *Anthropological Science*, *107*(2), 141-188.

Pollick, A. S., & de Waal, F. B. (2007). Ape gestures and language evolution. *Proc Natl Acad Sci U S A*, *104*(19), 8184-8189. <https://doi.org/10.1073/pnas.0702624104>
